# The candidate perspective of the clinical competency test (CCT) of the MICGP examination: a mixed-methods study

**DOI:** 10.3399/bjgpopen18X101605

**Published:** 2018-09-05

**Authors:** Tony Foley, Kathleen McLoughlin, Elaine K Walsh, Paul Leggett, Muríosa O'Reilly, Molly Owens, Aisling A Jennings

**Affiliations:** 1 Lecturer, Department of General Practice, University College Cork, Cork, Ireland; 2 Co-Convener of the CCT Exam, Examination Committee of the Irish College of General Practitioners, Irish College of General Practitioners, Dublin, Ireland; 3 Research Fellow, Department of General Practice, University College Cork, Cork, Ireland; 4 Lecturer, Department of General Practice, University College Cork, Cork, Ireland; 5 Co-Convener of the CCT Exam, Examination Committee of the Irish College of General Practitioners, Irish College of General Practitioners, Dublin, Ireland; 6 MICGP Exam Manager, Examination Committee of the Irish College of General Practitioners, Irish College of General Practitioners, Dublin, Ireland; 7 Chair of the MICGP Exam, Examination Committee of the Irish College of General Practitioners, Irish College of General Practitioners, Dublin, Ireland; 8 Clinical Research Fellow, Department of General Practice, University College Cork, Cork, Ireland

**Keywords:** general practice, family practice, education, training, assessment, clinical competency, postgraduate education

## Abstract

**Background:**

The clinical competency test (CCT) was introduced by the Irish College of General Practitioners (ICGP) in 2015. Similar to the clinical skills assessment (CSA) of the Membership of the Royal College of General Practitioners exam (MRCGP), the CCT is a modified objective structured clinical examination (OSCE).

**Aim:**

The aim of this study was to evaluate the MICGP CCT from the candidates' perspective, to gain an insight into their views of its fairness, relevance, and acceptability.

**Design & setting:**

This mixed-methods study was conducted with GP registrars in Ireland.

**Method:**

The study was conducted in two phases. Firstly, focus groups were conducted with participants who had previously undertaken the CCT to explore their experience of the CCT. Secondly, findings from the focus groups informed the development of an online questionnaire, which was sent to all GP registrars who completed the CCT in the 2017 summer sitting.

**Results:**

Two focus groups were held with a total of nine participants. Following this, the online questionnaire was emailed to 134 registrars. Of these, 83 registrars completed the questionnaire in full. Registrars reported that the CCT is a fair exam and is relevant to daily general practice. They considered the exam to be a comprehensive assessment that has a positive educational impact. However, they were challenged by time restrictions, and found it financially and emotionally stressful.

**Conclusion:**

This is the first study to evaluate the candidate’s perspective of an exiting GP membership exam in the UK or Ireland. The CCT is well-regarded by registrars. The study results will help to inform the future development of the CCT exam.

## How this fits

This study evaluates the CCT, the new exiting exam for GP registrars in Ireland, from the perspective of the candidates. The CCT is similar in structure to the MRCGP exiting exam, the CSA, being a modified OSCE. No previous research has evaluated the CSA from the perspective of the candidate. Furthermore, this study adds to existing international literature on evaluation of OSCE style examinations.

## Introduction

The goal of assessment in medical education is the development of reliable measures of clinical performance that also have a formative role and can drive students' learning.^1^ To this end, the CCT was introduced by the ICGP in 2015, representing a significant change in assessment modality for their exiting membership examination, the MICGP. Together with the modified essay question exam and the clinical knowledge test, which is a single best answer test, the CCT now forms part of an integrated, triangulated assessment.^2^ In addition to these formal summative assessment components of the MICGP, registrars also undertake informal workplace-based assessment with their GP trainers. The MICGP is the end point qualification for specialist training in general practice in Ireland and successful completion is needed to practice independently as a GP.

The CCT aims to assess the competence of general practice registrars when dealing with situations that may arise in everyday general practice. It ‘seeks to examine the knowledge, clinical skills and attitudes displayed by GP registrars as they manage patients presenting in a simulated GP surgery’.^2^ Similar in structure and format to the CSA of the MRCGP,^3^ the CCT is a modified OSCE. Candidates are assessed conducting GP consultations with actors. The scenarios for the ‘mock’ consultations are carefully developed to ensure they reflect the content and variety of typical GP consultations. The reliability of an OSCE exam is contingent on careful sampling across clinical content and requires an appropriate number of stations.^4^ Additionally, the reliability increases with increasing testing time.^5^ Informed by this, the CCT exam is comprised of 13 10-minute stations, with clinical content drawn from across the curriculum of GP training.

Concerns over the poor validity and reliability of other clinical assessment formats led to the introduction of OSCE-style exams,^6^ which have now been widely adopted by post-graduate training bodies. Research has reported that these exams are positively viewed by students and teachers and can motivate learning.^7^ Furthermore, this examination format allows a broad range of skills to be assessed^8^ and has been shown to have a high level of reliability and validity.^9^ However, evidence suggests that there are also negatives associated with OSCEs. Students rarely receive detailed, station-by-station verbal feedback to enable them to identify their strengths and weaknesses.^10^ The validity of OSCEs has been questioned, regarding the artificial context in which the exam takes place, and concern has been raised that OSCEs are as much about performance as they are about content.^11^ Adverse educational impacts on learners have also been reported. For instance, for OSCE preparation, students may avoid seeing real patients, in favour of exam practice with colleagues.^12^ Student anxiety is frequently cited as a further disadvantage.^13^ In addition, from a faculty perspective, the organisation of OSCE exams is complex, costly, and logistically challenging.^14^

From a general practice perspective, the CSA of the MRCGP has been extensively investigated, in particular focusing on ethnicity,^15^ sex,^16^ examiner bias,^17^ and training of role-players.^18^ However, a recent integrative review of the literature on the MRCGP CSA published by the authors^19^ identified the lack of published research on GP registrars’ views of these types of exams, despite the evidence that student feedback is an important source of evaluation evidence.^20^

The aim of this study was to evaluate the MICGP CCT from the candidates' perspective, in order to gain an insight into their views of its fairness, relevance, and acceptability.

## Method

### Study design

This was a mixed-methods study which employed an exploratory design.^21^ The study began with a qualitative data collection phase in the form of focus groups. The results of this qualitative analysis then informed a subsequent quantitative phase involving an online questionnaire.^22^

### Focus groups

No previously validated questionnaire was identified in the literature, therefore, in order to inform the development of an appropriate and robust questionnaire, focus groups were conducted with GP registrars who had previously sat the CCT. A topic guide for use in the focus groups was developed by the research team. The key areas of this interview guide were informed by literature on examination design and evaluation.^20^ The topic guide for the focus groups is available from the authors on request.

GP registrars who had experience of sitting the CCT were recruited from the Cork GP vocational training programme in the southern region of Ireland. One of the research team, an academic GP with previous experience in focus group facilitation, conducted both focus groups. The focus groups were audio-recorded and transcribed verbatim. Transcripts were anonymised, uploaded onto NVivo (version 10) software, and thematically analysed by two researchers, in accordance with the approach outlined by Braun and Clarke.^23^

### Questionnaire

Focus group findings, together with items adapted from previously used questionnaires to assess students’ views of OSCE examinations^24,25^ were used to develop a 50-item online questionnaire. The questionnaire (available from the authors on request) was divided into seven subsections (Box 1) and consisted of a series of statements with 5-point Likert-style responses. Furthermore, there was a section where free text responses were requested. This questionnaire was piloted with three GPs who had previously undertaken the CCT but had not participated in either focus group. No modifications were required. The online survey was facilitated by the LIME Survey platform:^26^ a link to the survey was sent to eligible participants, with a reminder email sent 7 days later.

The survey was distributed via email by the ICGP to a census sample of CCT candidates 2 days after sitting the CCT, in May 2017. A reminder email was sent 1 week later. No exclusion criteria were applied. Email was considered to be the most acceptable and resource-efficient way of distributing the questionnaire. Quantitative data from the survey were exported to SPSS (version 23) for analysis. Free-text comments from the survey were independently thematically analysed by two researchers.

## Results

### Focus groups

Two focus groups were held in March 2017. The first focus group (*n* = 5, duration 43 minutes) was conducted with GP registrars all of whom were in their final year of training and had completed the CCT the previous year. The second focus group (*n* = 4, duration 37 minutes) was conducted with GPs who had recently graduated from the vocational training programme. The GPs who participated in the second focus group were based in different geographical locations. As a result, due to the logistical challenges of a face-to face meeting, the second focus group was conducted via teleconference. The main findings from the focus groups are presented with supporting quotations in Box 2.

**Box 1.** The seven subsections of the online questionnaire

Candidate demographics Perception of clinical competency test attributes Attitude toward clinical competency test environment and exam set-up Attitude toward performance quality Perception of educational impact Perceived reliability and validity General comments

**Box 2.** Themes from focus groups with supporting quotes

Theme	Supporting quotes (focus group number, participant number)
Fairness and relevance of the exam	*‘So yeah, I thought it was all very fair and representative of real life, yeah.'* (FG1,P5) *‘It is basically examining you on a morning surgery ... ’* (FG2, P2) *'I think just working every day is good experience in itself, you know ... '* (FG1, P3) *' ... it was realistic ... and you know it was fair.'* (FG1, GP2)
Exam preparation	*‘You learn from people you study with too, you pick up on things, on how they consult, which is nice.’* (FG2, P3) *' ... on the day having practiced to time and having practiced the role-plays as much as possible, after the first two or three patients I felt personally the nerves went and you kind of just got in to it because you are used to doing it ... '* (FG2, P2) *' ... practicing with each other in groups is better than one to one with your trainer because they are out of training and they wouldn’t have done the exams for a few years whereas we are in the zone, so to speak ... '* (FG1, GP5)
Organisation of the exam	*‘It was like clockwork it was very well run. They did a very good job ... ’* (FG2, P1) *' ... the buzzers going and everyone like a military, out and in.*' (FG1, P1) *' ... really really well timed, it was really organised when I found it, like, it all ran perfectly ...* ' (FG1, P5)
Examiners	*'I thought they were very discreet, I barely noticed them … They were like ghosts just coming in and out and I think that just allowed us to focus on the task at hand ... ’* (FG1, P2) *'I barely noticed them ...* ' (FG1, P3) *'[The examiners were] totally just stone-faced*' (FG2, P3)
Actors	*‘You genuinely would just think that they were just patients sitting in front of you and then after two or three you just get in to that and accepted that they were who they said they were.’* (FG1, P2) *'They were really good at their role.'* (FG1, P3)
Exam expense	*'You can see where the money went there ... ’* (FG1, P3) *'I think it is outrageous that we don’t get fully refunded, that a good chunk of it has to come from our own pocket’* (FG1, P1)
Clinical impact	*'Afterwards, even though I was nervous about the result, I felt like I was better for doing the exam ... ’* (FG2, P3) *'It’s like your driving test, isn’t it? Some habits stay, but I definitely have kept on some of it yeah.'* (FG1, P2)
Feedback	*'It would have been nice to see how you had performed with this. I think it would have been nice to get some feedback. Passes are very helpful but it doesn’t tell you how well you did really.'* (FG1, P5) *'You know other exams that you do, you tend to bury and forget afterwards but of all the exams I have done this one, I would have loved to have known. You just get this big blank page of “pass” and that’s great and you are delighted that you passed, but you would love to know how you did in the different stations. Of all the exams we do this is literally what we are doing day to day for the rest of our lives.'* (FG1, P2) *'When we got the results it would have been nice to know what your mark was and just a little comment about how you did. It should just be a bit more transparent. Like, I presume they give feedback if somebody fails it. If you work hard at something, you know, you’d like to know how you did or how you could improve ... '* (FG2, P3)

### Questionnaires

All 134 candidates who participated in the CCT exam in the summer of 2017 were invited to participate in this study. A total of 94 responses were received; 11 were substantially incomplete and were excluded from the analysis. Therefore, 83 questionnaires were included in the final analysis, giving an effective response rate of 62%. Demographic information on study participants is outlined in Table 1.

**Table 1.** Candidate demographics

Variable		Frequency, *n* (%)
Sex	Female Male	60 (72) 23 (28)
Age, years	25 26–30 31–35 36–40 41–45 46–50	1 (1) 36 (43) 28 (34) 11 (13) 5 (6) 2 (2)
Location in which undergraduate medial training completed	Ireland UK Hungary Latvia Poland Pakistan Not specified	72 (87) 5 (6) 1 (1) 1 (1) 1 (1) 1 (1) 2 (2)
English as a first language	Yes No	78 (94) 5 (6)

### Fairness and relevance of the CCT

Over 75% of candidates who completed the online survey agreed that the exam cases were relevant to daily general practice, and were fair. While 94% of survey responders agreed that the exam covered a wide knowledge base, just over half (53%) agreed that the CCT covered a range of physical examination skills.

### Exam preparation

The majority of responders (86%) believed that practice sessions with their peers best prepared them for the CCT. In addition to peer-group learning, participants also used online resources to support their learning, with 86% of responders reporting that websites external to the ICGP website were useful in preparing for the CCT exam. However, tutorials provided by GP vocational training schemes and GP trainers were considered less useful.

### Organisation of the CCT

The majority of responders (90%) agreed that the CCT examination was well organised and ran smoothly. Invigilators were generally considered to be helpful (89%), however a minority of candidates (12%) felt that examiners could be more helpful in guiding them through the CCT process. This finding also emerged in the qualitative analysis of the free-text comments:

*‘Some examiners were friendly, others were very blunt and unfriendly, they wouldn't even look at you. This is somewhat off-putting. They could at least acknowledge your existence. Some of them never even said goodbye ... ’*

### Authenticity of the CCT

Sixty-nine percent of participants found the actors to be realistic, akin to real patients sitting in front of them. However, candidates did comment that the actors appeared to have been asked to *'hold back*' at times and not share information unless asked. Candidates suggested that in real clinical practice patients were more likely to share information more readily.

### Stress

Ninety percent of responders agreed that the CCT was stressful and 74% agreed that it was an intimidating method of assessment. Stress emerged as a major theme in the qualitative analysis of the free-text comments, with a number of participants reflecting on the stress the exam caused them. Waiting for the exam to commence was identified as a particular source of stress by a number of candidates. For many others, the biggest stressor was the need to complete paperwork within the allotted 10 minutes.

There was a perception that the level of stress induced by the exam may *'negatively impact on candidate performance*'. However, other candidates reflected that although the exam was stressful, this actually increased the authenticity of the exam as a real-life session in general practice is often stressful:

*‘I found the situation to be very challenging and intimidating at times. In reality, these are the real challenges of general practice ... ’*

### Cost of the CCT

The majority of candidates (68%) felt that the cost of sitting the CCT exam was unreasonable. Eighty-nine percent agreed that the full cost should be reimbursed and not just partially refunded, as it currently is. Dissatisfaction with the cost of the exam and the process of reimbursement also emerged as a theme in the qualitative analysis of the free-text comments:

*‘The cost to the candidate is too much upfront, waiting for reimbursement can be frustrating and seems pointless. Money going round in circles essentially ... ’*

### Timing of cases

There was negative feedback from the majority of candidates (66%) regarding the 10 minutes allocated for each case. In the free-text comments participants further elaborated on the reasons why they felt the time provided was insufficient. Participants felt that the need to include written prescriptions and referral letters within this allocation was unrealistic and not reflective of their current clinical practice:

*‘In real life many of us write our referral letters after the consultation or later in the day when we have more time, so that we can keep up with appointments ... ’*

In Ireland, the standard consultation time varies from practice to practice but typically ranges from 10–15 minutes. A number of candidates suggested increasing the allotted time to 12–15 minutes per case, as this would be *‘more comparable to our day to day practice to wrap up, follow up, and safety netting ... ’*

### Educational impact

For most candidates there was a perception that undertaking the CCT has improved their consultation skills (80%) and their confidence as a GP registrar (68%), and that preparing for it highlighted areas of weakness in skills and knowledge (63%).

## Discussion

### Summary

These findings indicate that candidates deem the CCT to be a fair, acceptable examination, relevant to daily practice; however, concern was raised about the financial and psychological impact of the exam. Candidates found that the examination was run efficiently, with accomplished role-players and unobtrusive, if not overly helpful, examiners. Candidates were challenged by time management restrictions and found the assessment to be stressful and expensive. Candidates considered the CCT to be a comprehensive assessment of knowledge and consultation skills, though perhaps not of physical examination skills. Despite this, there was broad acceptance that participation in the CCT had a positive educational impact.

### Strengths and limitations

For pragmatic reasons, the second focus group was held via teleconference. While teleconferencing is recognised as a feasible method of data collection for interviewing geographically dispersed, busy individuals,^27^ facilitators are unable to pick up and respond to potentially valuable non-verbal cues.^28^ Interaction within the group is a key aspect of a focus group, to foster rich discussions and sharing of experiences. A focus group via teleconference has the potential to limit this interaction. However, the participants of the focus group were all part of the same GP training class. As they were a pre-existing group, they enjoyed a comfort and familiarity with each other which facilitated discussion and the ability to challenge each other comfortably.^29^ The authors feel that this overcame some of the potential limitations of using teleconferencing to conduct the second focus group.

The study was conducted among a single group of CCT candidates. In addition, responders completed the questionnaire the week after undertaking the CCT and, though anonymous, may have given socially desirable responses before they had received their examination results. However, it was felt that by sending the questionnaire out in the week immediately following the exam, candidates were more likely to respond as they were likely to be still reflecting on the exam. Sending out the questionnaire several weeks later might have negatively impacted on the response rate and on the accuracy with which the candidates recollected specific elements of the exam. Additionally, sending out the questionnaire to the candidates when they were still unaware of whether they passed the exam also helped to avoid any positive or negative bias towards the exam. Some members of the research team are also members of the exam committee of the ICGP. Cognizant of this, the authors attempted to minimise bias in the data collection and interpretation. The focus group data collection was performed by a researcher who does not have a formal connection with the exam, while data analysis was independently performed by two researchers. Nevertheless, participants remained aware that this study was being undertaken by members of the exam committee and so may have been influenced to give positive accounts of the CCT.

### Comparison with existing literature

Candidates’ positive view of the CCT echoes findings from reviews of students’ perception of other OSCE-style exams, which found that OSCE examinations are generally well-received by students,^7^ and are deemed to be authentic and comprehensive.^25^ Furthermore, candidates emphasised that the CCT had stimulated learning that was relevant to their daily practice, in agreement with reviews of the educational impact of other OSCEs.^14^

In terms of preparation for the CCT, candidates valued small-group, peer-learning using role-play. Similar findings were reported in a review of how candidates like to prepare for the UK equivalent, the CSA, which also found that valuing group study made a candidate 1.4 times more likely to pass the CSA at their first sitting.^30^ However, candidates were not as positive regarding the value of in-practice tutorials for the CCT with their GP trainers, again in agreement with previous research on the CSA.^30^ This warrants consideration of how much Irish GP trainers know about the CCT, which is a relatively new exam. Interestingly, a survey in England in 2013, 6 years after the introduction of the CSA, found that educational supervisors lacked knowledge about how the CSA was marked.^31^ Candidates’ preference for personalised feedback on their performance emerged as a particularly significant finding in both focus groups and echoes findings from other studies that indicate that students want to know specifics about how they have performed.^32^ Currently, only candidates that fail receive detailed personalised feedback in the CCT.

A notable finding from this study is the negative impact candidates feel in terms of stress and expense. Candidates clearly found the assessment to be stressful, a finding that concurs with previous studies of other OSCEs.^7,13^ In particular, they expressed concern regarding time management. However, some candidates considered this stress to be reflective of daily practice, adding to the validity of the exam. This is in line with the findings of previous studies.^33^ While a degree of exam stress is inevitable, efforts need to be made to minimise unnecessary sources of anxiety that may affect candidate performance.

There was broad dissatisfaction among candidates regarding the personal financial cost of sitting the CCT. Currently it costs candidates €1300 to sit the CCT, while a refund of €900 is available to candidates after their first sitting of the exam. For many, these expenses appear to be a source of significant stress. The considerable expense of running OSCEs, relative to other types of examinations, has previously been identified as a significant limitation of this type of examination.^7^

### Implications for research and practice

While the CCT was generally considered a fair, acceptable examination, relevant to daily practice, it is also evident that candidates feel that there are significant financial and emotional stresses involved. Assessment of performance in an examination will unavoidably induce stress for candidates. Clinical skills exams, such as the CCT, afford the opportunity to assess candidates’ performance under these stressful situations. Many candidates accepted that a certain amount of stress was inevitable and even saw it as further evidence of the authenticity of the exam. However, they felt there were areas of the exam process where stress could be reduced. The implication of this finding is that efforts to minimise unnecessary stress need to be considered, by making the structure and process of the exam as clear and explicit as possible. Further supporting material for CCT candidates needs to be developed for these critical areas. Regarding the financial burden, OSCE style exams are expensive to run and the model of partial reimbursement on successfully passing the exam is similar in other postgraduate training bodies such as the Irish postgraduate medical exams. However, it is possible that transparent explanations of the different costs associated with the exam may help to justify to candidates the considerable costs involved. The satisfaction of the GP registrars regarding their experiences with the post-graduate examinations they complete is important; it helps to foster pride in the qualification received. Future research should focus on other perspectives of the fairness and relevance of the CCT. This study highlighted that GP registrars did not appear to value GP trainer preparation for the CCT exam. Therefore, triangulating the views of GP trainers with other relevant stakeholders, such as CCT examiners and CCT actors, would be an interesting area for future research. This study also highlights other areas for future research including exam stress, financial burden, and whether GP registrar satisfaction influences recruitment into training.

To the best of the authors' knowledge, this is the first evaluation of candidates’ perceptions of an exiting professional GP clinical skills examination that has been conducted nationally or internationally. The findings of this study have been reported to the ICGP and the study results will help to inform the future development of the CCT exam.
